# Clinical associations and prognosis in Asian and European patients with symptom‐controlled atrial fibrillation: Insights from two prospective registries in Europe and Asia

**DOI:** 10.1111/eci.70086

**Published:** 2025-06-09

**Authors:** Wee Siong Teo, Manlin Zhao, Tommaso Bucci, Steven Ho Man Lam, Hongyu Liu, Yang Chen, Giuseppe Boriani, Hung‐Fat Tse, Tze‐Fan Chao, Gregory Y. H. Lip

**Affiliations:** ^1^ Department of Cardiology National Heart Centre Singapore Singapore; ^2^ Liverpool Centre for Cardiovascular Science at University of Liverpool Liverpool John Moores University and Liverpool Heart and Chest Hospital Liverpool UK; ^3^ Department of Cardiology Beijing Anzhen Hospital, Capital Medical University, National Clinical Research Centre for Cardiovascular Diseases Beijing China; ^4^ Department of Clinical Internal, Anaesthesiologic and Cardiovascular Sciences Sapienza University of Rome Rome Italy; ^5^ Department of Medicine and Therapeutics The Chinese University of Hong Kong Sha Tin New Territories Hong Kong; ^6^ Department of Cardiovascular Medicine The Second Affiliated Hospital, Jiangxi Medical College, Nanchang University Nanchang Jiangxi China; ^7^ Department of Cardiovascular and Metabolic Medicine Institute of Life Course and Medical Sciences, University of Liverpool Liverpool UK; ^8^ Cardiology Division, Department of Biomedical, Metabolic and Neural Science Italy University of Modena and Reggio Emilia, Policlinico di Modena Modena Italy; ^9^ Division of Cardiology, Department of Medicine, School of Clinical Medicine Queen Mary Hospital, The University of Hong Kong Hong Kong China; ^10^ Institute of Clinical Medicine and Cardiovascular Research Centre, National Yang Ming Chiao Tung University Taipei Taiwan; ^11^ Division of Cardiology, Department of Medicine Taipei Veterans General Hospital Taipei Taiwan; ^12^ Department of Clinical Medicine Aalborg University Aalborg Denmark; ^13^ Department of Cardiology, Lipidology and Internal Medicine Medical University of Bialystok Bialystok Poland

**Keywords:** anticoagulation, asymptomatic, atrial fibrillation, ethnic differences, rhythm control

## Abstract

**Background:**

Clinical associations and prognosis of patients with symptom‐controlled AF (scAF) remain poorly understood.

**Methods:**

We analysed data from the Asian‐Pacific Heart Rhythm Society and EURObservational Research Programme registries. Based on the European Heart Rhythm Association (EHRA) score, patients were classified as scAF (EHRA I or II) or symptomatic AF (EHRA III or IV). Clinical characteristics were examined by logistic regression, and prognosis was assessed by Cox models. The primary outcome was composed of all‐cause death and major cardiovascular events. Interaction analyses were performed to investigate ethnic differences.

**Results:**

Among 13,577 AF patients (mean age 69.0 ± 11.6 years; 38.7% female), 11,470 (84.5%) had scAF. Asians were more likely to be scAF, characterised by younger age and lower cardiovascular burden compared to Europeans. Diabetes mellitus was significantly associated with scAF only in Asians (adjusted odd ratio [aOR] 1.43, 95% confidence interval [CI] 1.03–2.04, *p*_interaction_ = 0.021). The associations with hypertension (aOR 1.29, 95% CI 0.98–1.70, *p*_interaction_ = 0.004) and prior ischemic stroke (aOR 1.75, 95% CI 0.96–3.58, *p*_interaction_ = 0.045) were more evident in Asians.

Patients with scAF showed a notable association with increased likelihood of using vitamin K antagonists (aOR 1.19, 95% CI 1.07–1.33), which was more prominent in Asians. In both Asians and Europeans, scAF was associated with reduced rhythm control management. Compared to non‐scAF, European patients with scAF had a reduced risk of the composite outcome, but the association was non‐significant in Asians (*p*_interaction_ = 0.594).

**Conclusion:**

Asians and Europeans with scAF demonstrate clinically relevant differences in terms of overall prevalence, related risk factors, and clinical management.

## Graphical abstract

Symptom‐controlled atrial fibrillation (AF), defined as asymptomatic or with mild symptoms according to the European Heart Rhythm Association (EHRA) score, was more common among Asians than in Europeans. In Asians, comorbidities of diabetes, hypertension, and prior ischemic stroke increase the likelihood of being scAF. Patients with scAF were more likely to be prescribed vitamin K antagonists, and this tendency was more prominent in Asians. Patients with symptom‐controlled AF were less likely to receive rhythm control treatment, without ethnic differences observed.

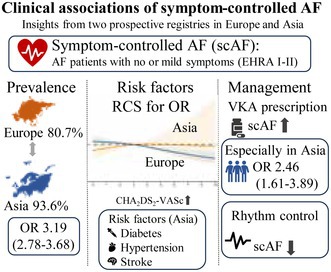

## 1 INTRODUCTION

Atrial fibrillation (AF) is the most common arrhythmia and is rapidly increasing in Asia and Europe due largely to increasing lifespan and a rapidly aging population.^1^, ^2^ Its importance lies in the association of AF with significant morbidities such as stroke, heart failure (HF), dementia, and increased mortality.^1^

AF‐related symptoms are a key driver for medical consultation and a crucial consideration in treatment due to their possible impact on quality of life, clinical management and long‐term prognosis.^3^, ^4^, ^5^ The symptomatic status has been considered the main determinant for rhythm control procedures with potential advantages in terms of risk of adverse events, given recent evidence showing the superiority of this approach compared to rate control.^6^ The current assessment of AF‐related symptoms is based on their impact on quality of life, as classified by the European Heart Rhythm Association (EHRA) score. Symptom‐controlled AF (scAF) is defined as having an asymptomatic status (EHRA I) or mild symptoms (EHRA II), whereas symptomatic AF (sAF) is characterised by severe symptoms (EHRA III) or disabling symptoms (EHRA IV).^5^, ^7^

However, it should be noted that the absence of severe or disabling symptoms does not imply reduced risks of adverse outcomes. Previous studies have shown that the clinical prognosis of asymptomatic AF can be similar or even worse compared to symptomatic AF (sAF).^8^, ^9^, ^10^, ^11^ Additionally, a non‐negligible proportion of AF patients may present for the first time with an ischemic stroke or HF, without prior symptoms.^12^, ^13^ This suggests the presence of a considerable proportion of AF patients in an inherently asymptomatic status, which cannot be utilised for risk stratification.

In this study, utilising data from the two large observational registries from the Asia‐Pacific and European regions, we aimed to explore the clinical characteristics and prognosis of AF patients with scAF and to compare potential differences between Asians and Europeans.

## 2 METHODS

The analyses were conducted based on the Asian‐Pacific Heart Rhythm Society (APHRS) and EURObservational Research Programme (EORP) registries. The EORP registry involved 250 centres across 27 European countries between 2013 and 2016. The APHRS registry included AF patients from five East Asian regions: Hong Kong, South Korea, Japan, Singapore, and Taiwan, from 2015 to 2017. Both cohorts consecutively enrolled inpatients and outpatients from tertiary, secondary, and general hospitals. The two registries share similar protocols, which were approved by local ethics committees, and all patients provided written informed consent.

### 2.1 Study population

Diagnosis of AF was based on electrocardiogram (ECG) within the past 12 months. Based on the EHRA score at baseline, patients were subdivided into scAF (EHRA I or II) and sAF (EHRA III or IV). Patients with missing values of EHRA score and the primary outcome were excluded.

### 2.2 Study Outcomes

As per the original design of the two registries, patients enrolled in the EORP registry were followed for 2 years, while those recruited in the APHRS registry were followed for 1 year. We defined the primary outcome as a composite of all‐cause death and major adverse cardiovascular events (MACE). MACE included cardiovascular death, acute coronary syndrome (ACS), or any thromboembolic events (TE). The secondary outcomes in our analysis were each component of the composite endpoint (all‐cause death, MACE, cardiovascular death, ACS, TE) and major bleeding (MB). The incidence of adverse outcomes was collected by the investigators during the follow‐up.

### 2.3 Treatment patterns

The treatments, including the use of oral anticoagulants (OACs), the type of OACs (vitamin K antagonists [VKAs] or novel oral anticoagulants [NOACs]), and administration of a rhythm control strategy, were documented at discharge or after consultation. The type of OACs was only defined for patients who were prescribed OACs. Different types of NOACs, including dabigatran, rivaroxaban, apixaban and edoxaban, were identified among patients taking NOACs. Patients who received a rhythm control intervention such as electrical or pharmacological cardioversion, catheter ablation, or were prescribed an antiarrhythmic drug (Class Ia, Class Ic, Class III) were included in the ‘rhythm control’ group. All the other patients were considered as not receiving rhythm control.

### 2.4 Statistical analysis

Continuous variables were presented as median with interquartile ranges (IQR), and categorical variables were presented as percentages. Differences between symptom‐controlled and symptomatic patients were examined by Mann–Whitney *U* test for continuous variables and chi‐square test for categorical variables.

Multivariable logistic regression was utilised to explore: (1) clinical factors associated with the scAF, (2) the use and specific type of OACs, (3) specific types of NOACs, and (4) the likelihood of receiving rhythm control treatment. All models were adjusted for age, sex, paroxysmal AF, HF, diabetes mellitus (DM), hypertension, vascular diseases (VAS), chronic kidney disease (CKD), ischaemic stroke, cancer, and ethnicity. Similar multivariable logistic models were performed separately for Asian and European groups, excluding ethnicity as a covariate.

Associations between CHA_2_DS_2_‐VASc score and probability of scAF among Asians and Europeans were evaluated by restricted cubic spline curves with three knots based on unadjusted logistic regression models.

Univariable and multivariable Cox regression models were used to evaluate the risk of primary and secondary outcomes in scAF patients compared to sAF patients, with adjustment for age, sex, paroxysmal AF, HF, DM, hypertension, VAS, CKD, ischemic stroke, cancer, and OACs.^14^ For sensitivity analysis, we specifically applied the same Cox models to investigate the association between asymptomatic AF (EHRA I) and clinical outcomes.

Interaction analysis was additionally performed in both logistic and Cox models to assess how ethnicity influenced risk factors, treatment including prescription of OAC, OAC type, and rhythm control strategy, along with clinical outcomes associated with scAF.

Missing values for the adjusted variables were imputed using an iterative imputation method (missForest) based on a random forest.^15^ The R package missForest (version 1.5) was used to perform imputation.

The threshold for statistical significance was *p* < 0.05. The hypothesis test was two‐sided. All analyses were performed with the R software (R Core Team (2024). R: A Language and Environment for Statistical Computing. R Foundation for Statistical Computing, Vienna, Austria. URL https://www.R‐project.org/).

## 3 RESULTS

### 3.1 Ethnic differences in baseline characteristics in the overall population

A total of 13,577 AF patients (mean age 69.0 ± 11.6 years; 38.7% female) were included in the analysis, with 3957 patients (29.1%, median age 69.0 years [IQR 61.0–77.0], 34.9% females) from the APHRS‐AF Registry and 9620 (70.9%, median age 71.0 years [IQR 63.0–77.0], 40.2% females) from the EORP‐AF Registry. The prevalence of scAF was higher in Asians than in Europeans (93.6% vs. 80.7%, *p* < 0.01) (Table S1).

Overall, 11,470 (84.5%) AF patients were scAF, exhibiting lower prevalence of comorbidities and proportions of patients with CHA_2_DS_2_‐VASc score ≥2 or HAS‐BLED score ≥3, compared to those with sAF. Patients with scAF had lower proportions of undergoing a rhythm control strategy, that is, undergoing catheter ablation or electrical cardioversion, and receiving AAD prescriptions compared to those with sAF (Table S2).

### 3.2 Ethnic differences in baseline characteristics associated with scAF

On multivariable logistic regression, Asians had higher odds of being scAF, compared to Europeans (adjusted Odd Ratio [aOR] 3.19, 95% Confidence Interval [CI] 2.78–3.68) (Table S3).

Among scAF patients, 3702 (32.3%) were Asians, with a younger age (median age 69.0 [IQR 61.0–77.0] vs. 71.0 [IQR 63.0–78.0] years, *p* < 0.01), lower BMI (24.7 [22.4–27.3] vs. 27.6 [24.8–31.1] kg/m^2^, *p* < 0.01), and fewer females (34.3% vs. 38.2%, *p* < 0.01), compared to Europeans. Asians with scAF had a higher proportion of paroxysmal AF, DM, and dementia, but a lower prevalence of HF, CAD, dyslipidaemia, CKD, and COPD (Table 1).

**TABLE 1:** Baseline characteristics for patients with symptom‐controlled AF.

	EORP(*n* = 7768)	APHRS (*n* = 3702)	*p* value
Age (year)	71.0 [63.0, 78.0]	69.0 [61.0, 77.0]	<0.001
Age ≥65 years (%)	5443 (70.1)	2439 (65.9)	<0.001
Female (%)	2969 (38.2)	1269 (34.3)	<0.001
BMI (kg/m^2^)	27.6 [24.8, 31.1]	24.7 [22.4, 27.3]	<0.001
HF (%)	2801 (36.4)	772 (21.1)	<0.001
CAD (%)	2004 (27.1)	708 (19.5)	<0.001
Hypertension (%)	4713 (61.2)	2280 (62.0)	0.445
DM (%)	1744 (22.6)	904 (24.8)	0.009
Lipid disorder (%)	3052 (41.0)	1403 (38.6)	0.014
Thromboembolic events (%)	894 (11.6)	420 (11.5)	0.857
Haemorrhagic events (%)	403 (5.2)	274 (7.5)	<0.001
CKD (%)	892 (11.6)	277 (7.5)	<0.001
Liver disease (%)	187 (2.4)	168 (4.6)	<0.001
COPD (%)	665 (8.6)	96 (2.6)	<0.001
Dementia (%)	88 (1.1)	68 (1.8)	0.003
Cancer (%)	158 (2.0)	78 (2.1)	0.876
CHA_2_DS_2_‐VASc score	3.0 [2.0, 4.0]	3.0 [1.0, 4.0]	<0.001
CHA_2_DS_2_‐VASc score ≥2	6206 (80.0)	2723 (73.6)	<0.001
HAS‐BLED score	1.0 [1.0, 2.0]	1.0 [1.0, 2.0]	<0.001
HAS‐BLED score ≥3	1304 (16.8)	510 (13.8)	<0.001
Paroxysmal AF (%)	1938 (25.4)	1524 (41.3)	<0.001
Persistent AF (%)	1796 (23.6)	1230 (33.3)	<0.001
OACs (%)	6720 (86.5)	3061 (82.7)	<0.001
VKAs (%)	3871 (49.8)	781 (21.1)	<0.001
NOACs (%)	2854 (36.8)	2280 (61.6)	<0.001
Rhythm control strategy at discharge (%)	2851 (36.7)	1075 (29.0)	<0.001
Catheter ablation (%)	353 (14.3)	467 (12.6)	0.054
Electrical cardioversion (%)	1223 (49.7)	78 (2.1)	<0.001
AAD prescription at discharge (%)	1903 (24.5)	870 (23.5)	0.253

Abbreviations: AAD, antiarrhythmic drugs; AF, atrial fibrillation; BMI, body mass index; CAD, coronary artery disease; CKD, chronic kidney disease; COPD, chronic obstructive pulmonary disease; DM, diabetes mellitus; HF, heart failure; NOAC, novel oral anticoagulants; OAC, oral anticoagulants; VKA, vitamin K antagonists.

**FIGURE 1:**
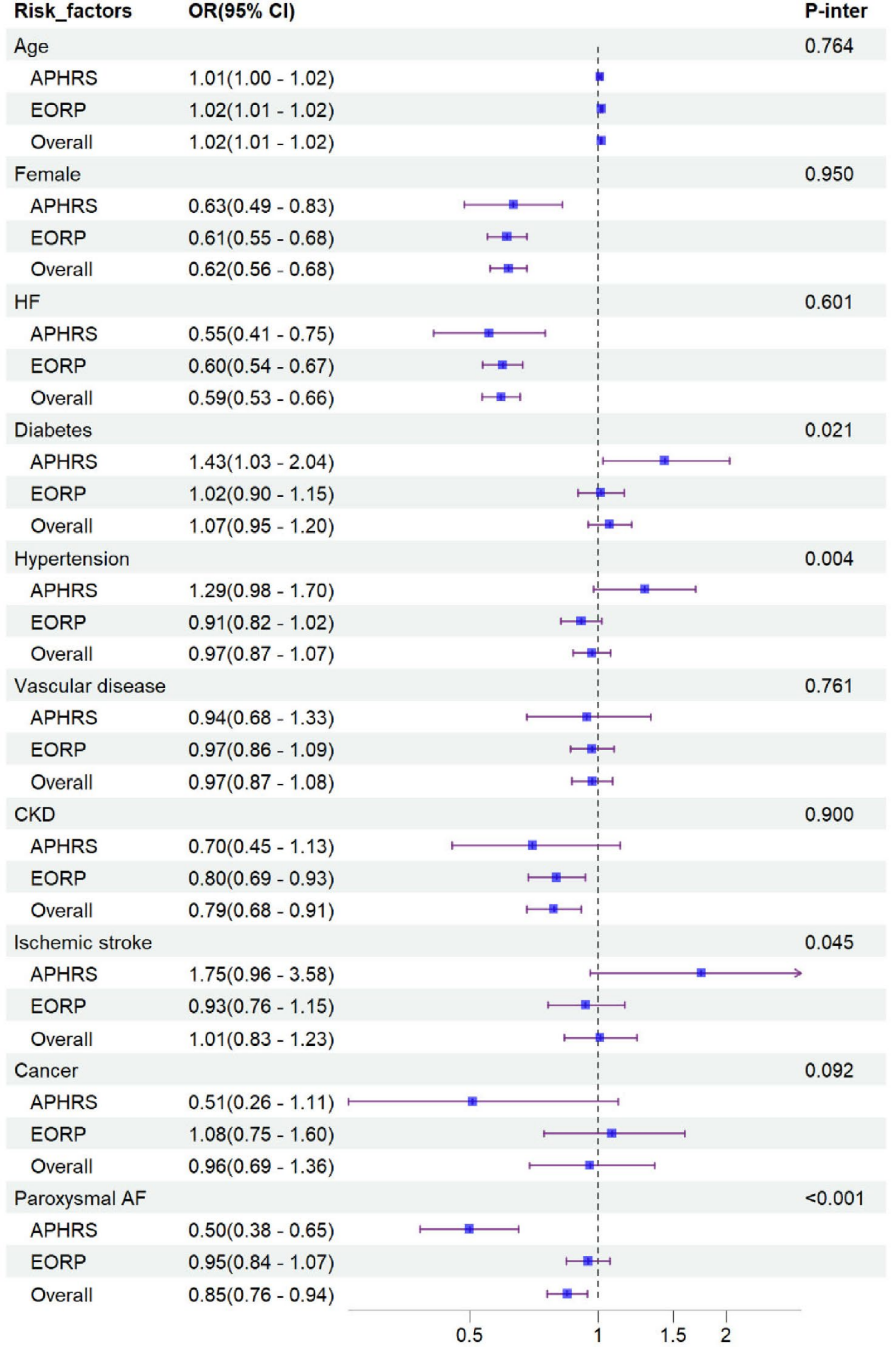
Clinical factors for symptom‐controlled AF in the two registries. Logistic models among overall population were adjusted for age, sex, paroxysmal AF, HF, DM, hypertension, vascular diseases, CKD, ischemic stroke, cancer, ethnicity. Logistic models performed in EORP and APHRS cohort separately were adjusted for age, sex, paroxysmal AF, HF, DM, hypertension, vascular diseases, CKD, ischemic stroke, cancer.

On multivariable logistic regression, advanced age was associated with an increased likelihood of scAF, whereas female sex, HF, CKD, and paroxysmal AF were related to lower risks of scAF (Figure 1).

When considering ethnicity separately, DM had a greater impact on scAF in Asians compared to Europeans (aORs 1.43, 95% CI 1.03–2.04 and 1.02, 95% CI 0.90–1.15 respectively, *p*_interaction_ = 0.021). The association between paroxysmal AF and a reduced likelihood of scAF was more evident in Asians than in Europeans (aORs 0.50, 95% CI 0.38–0.65 and 0.95, 95% CI 0.84–1.07 respectively, *p*_interaction_ <0.001). Similarly, hypertension and prior ischemic stroke exhibited more prominent impacts on increasing the likelihood of being scAF, which were not observed in Europeans (*p*_interaction_ 0.004 for hypertension and 0.045 for prior ischemic stroke) (Figure 1).

**FIGURE 2:**
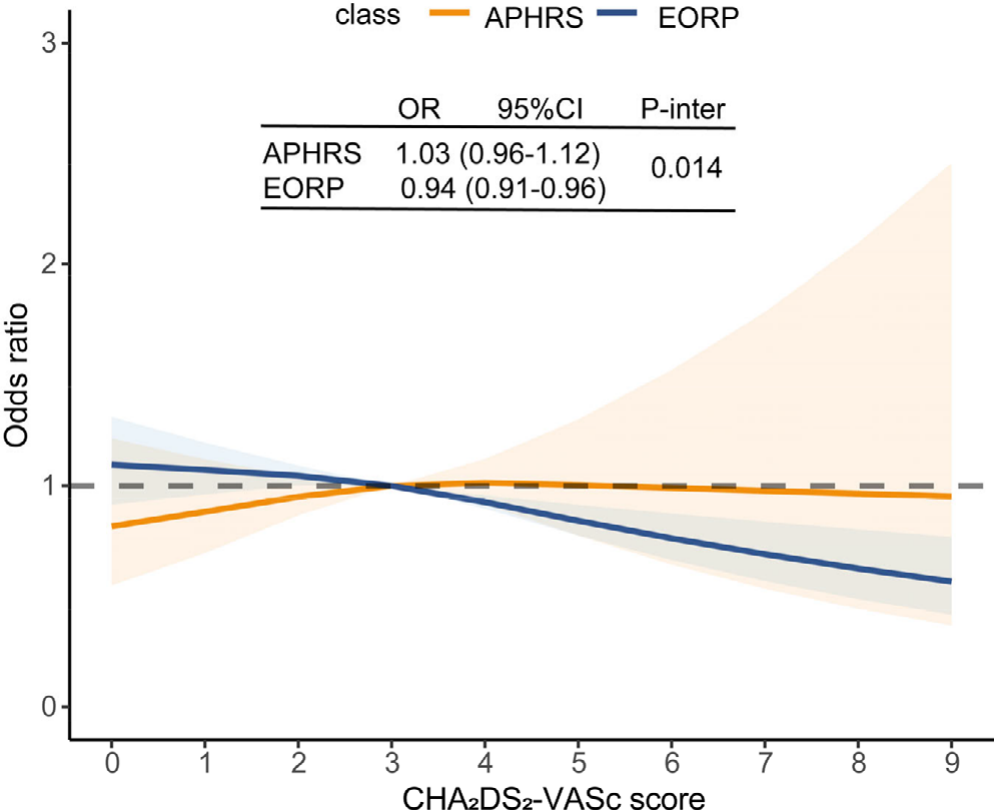
Association between CHA_2_DS_2_‐VASc scores and the probability for symptom‐controlled atrial fibrillation based on unadjusted logistic regression. *p* for interaction was between symptom‐controlled AF and ethnicity (Asians vs. Europeans).

The unadjusted probabilities for scAF based on CHA_2_DS_2_‐VASc score are shown in Figure 2. According to the restricted cubic spline curves, the probability of scAF in Asians increased with higher CHA_2_DS_2_‐VASc score, whereas the trend was reversed in Europeans. Though the relationship between CHA_2_DS_2_‐VASc score and scAF was nonsignificant in Asians, Europeans with elevated CHA_2_DS_2_‐VASc score were less likely to present with scAF (OR 1.03, 95% CI 0.96–1.12 and 0.94, 95% CI 0.91–0.96 respectively, *p*_interaction_ 0.014).

### 3.3 Ethnic differences in treatment patterns associated with scAF

**FIGURE 3:**
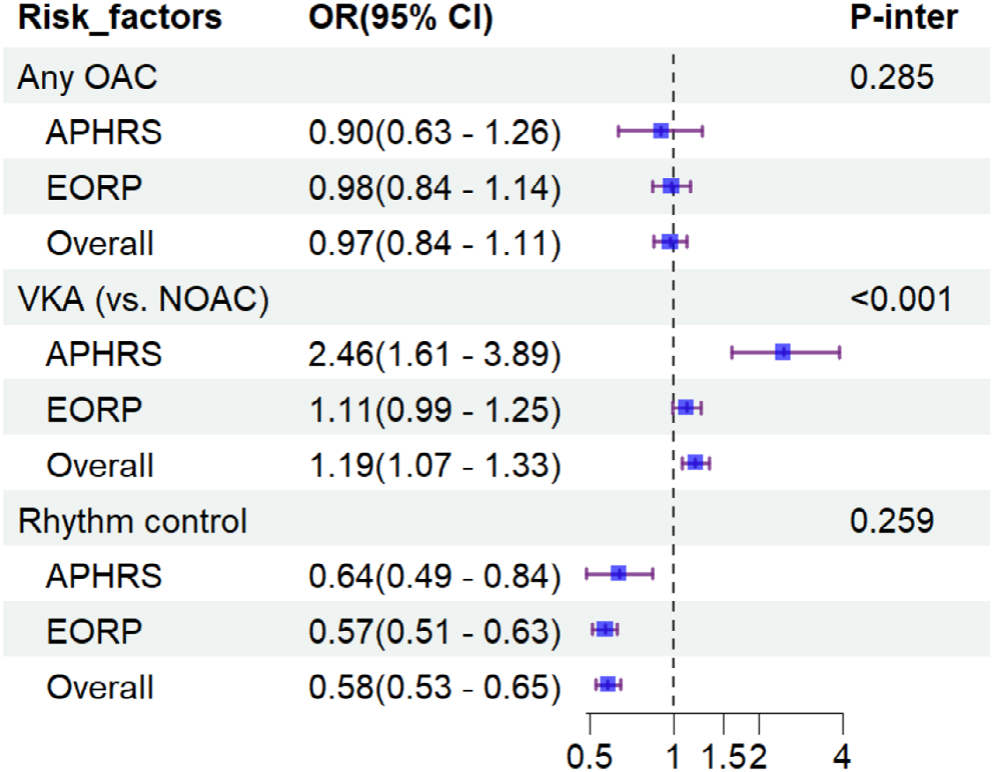
Adjusted logistic regression analysis investigating the association between symptom‐controlled AF and different AF management patterns. Logistic models among overall population were adjusted for age, sex, paroxysmal AF, heart failure, DM, hypertension, vascular diseases, chronic kidney disease, ischemic stroke, cancer, ethnicity. Logistic models performed in EORP and APHRS cohort separately were adjusted for age, sex, paroxysmal AF, heart failure, DM, hypertension, vascular diseases, chronic kidney disease, ischemic stroke, cancer. AF, atrial fibrillation; CKD, chronic kidney disease; DM, diabetes mellitus; HF, heart failure; NOAC, novel oral anticoagulants; OAC, oral anticoagulants; VKA, vitamin K antagonist.

In the entire population, no significant association was found between scAF and the use of OACs (aOR 0.97, 95% CI 0.84–1.11). In contrast, scAF was significantly associated with higher odds of using VKAs (aOR 1.19, 95% CI 1.07–1.33), and a lower likelihood of adopting a rhythm control strategy (aOR 0.58, 95% CI 0.53–0.65) (Figure 3). Associations between scAF and different types of NOACs were not significant (Table S4).

Regarding ethnic differences, the association between scAF and increased likelihood of VKAs prescription was more evident in Asians than in Europeans (ORs 2.46, 95% CI 1.61–3.89 and 1.11, 95% CI 0.99–1.25, respectively; *p*_interaction_ <0.001) (Figure 3). Among patients taking NOACs, those with scAF in Europe showed a notable prescription preference for edoxaban compared to Asians (ORs 5.09, 95% CI 2.10–16.74 and 0.65, 95% CI 0.45–0.95, respectively; *p*_interaction_ <0.001) (Table S4).

Among patients with scAF, Europeans had a significantly higher proportion of adopting rhythm control strategy (36.7% vs. 29.0%, *p* < 0.001) compared to Asians. Specifically, electrical cardioversion was more frequently performed in Europeans than in Asians (Table 1). Nevertheless, the association between scAF and rhythm control treatment was consistent in both groups (*p*_interaction_ = 0.259) (Figure 3).

### 3.4 Ethnic differences in adverse outcomes associated with scAF

During a median follow‐up of 701 [IQR 365–736] days, a total of 1477 (10.9%) cases of primary outcome occurred. Overall, patients with scAF showed lower incidence rates for the composite outcome, compared to those with sAF. Patients with scAF were significantly associated with reduced risks of the composite outcome (adjusted hazard ratio [aHR] 0.77, 95% CI 0.67–0.87), and several secondary outcomes, including all‐cause death, MACE, and cardiovascular death (Table 2 and Table S5).

**TABLE 2:** Results of multivariable cox regression analysis for the relationship between symptom‐controlled AF and outcomes of the study.

	scAF IR/1000 person years	sAF IR/1000 person years	HR	95% CI	*p* value	*p* for inter
Composite outcomes						
APHRS	40.6	48.2	0.93	0.49–1.75	0.820	0.594
EORP	72.0	94.0	0.79	0.70–0.91	0.001	
Overall	65.6	90.8	0.77	0.67–0.87	<0.001	
All‐cause death						
APHRS	28.6	35.7	0.92	0.44–1.95	0.831	0.596
EORP	49.2	65.0	0.78	0.67–0.91	0.002	
Overall	45.0	63.0	0.76	0.66–0.88	<0.001	
MACE						
APHRS	19.6	20.4	0.99	0.37–2.61	0.982	0.616
EORP	40.7	56.3	0.79	0.67–0.94	0.007	
Overall	36.4	53.8	0.75	0.64–0.89	0.001	
ACS						
APHRS	6.2	12.2	0.51	0.15–1.74	0.282	0.376
EORP	13.5	16.1	0.90	0.67–1.23	0.518	
Overall	12.0	15.9	0.83	0.61–1.12	0.209	
Cardiovascular death						
APHRS	6.6	4.0	1.85	0.23–15.24	0.566	0.320
EORP	17.3	26.8	0.73	0.57–0.93	0.011	
Overall	15.1	25.2	0.70	0.55–0.89	0.004	
Any TE						
APHRS	7.6	4.1	1.91	0.26–14.13	0.527	0.372
EORP	11.6	16.4	0.75	0.55–1.03	0.076	
Overall	10.8	15.6	0.74	0.55–1.01	0.057	
Major bleeding						
APHRS	11.8	20.6	0.51	0.19–1.38	0.183	0.290
EORP	11.2	11.0	1.05	0.73–1.51	0.805	
Overall	11.3	11.7	0.96	0.68–1.36	0.826	

*Note:* Adjusted for: age, sex, paroxysmal AF, heart failure, diabetes mellitus, hypertension, vascular diseases, chronic kidney diseases, ischemic stroke, cancer, oral anticoagulants.

Abbreviations: ACS, acute coronary syndrome; CI, confidence interval; HR, hazard ratio; IR, incidence rate; MACE, major adverse cardiovascular events; sAF, symptomatic atrial fibrillation; scAF, symptom‐controlled atrial fibrillation; TE, thromboembolic events.

When stratifying by ethnicity, the risks of primary and secondary outcomes were not related to scAF in Asians, whereas Europeans with scAF showed a reduced risk of composite outcome and several secondary outcomes, including all‐cause death, MACE and cardiovascular death, compared to sAF patients. No significant interaction effects were observed for Asians and Europeans regarding the effects on both primary (*p*_interaction_ 0.594) and secondary outcomes (Table 2 and Table S5).

### 3.5 Ethnic differences in adverse outcomes associated with asymptomatic AF

Associations between asymptomatic AF (EHRA I) and clinical outcomes were shown in Table S6. Asymptomatic AF was not significantly associated with composite outcome and secondary outcomes among the overall population and in the EORP cohort. For Asians, asymptomatic AF was significantly associated with elevated risks of thromboembolic events (aHR 3.03, 95% CI 1.03–8.90, *p*_interaction_ 0.024). Similar trends of increased risks in Asians were also observed for composite outcome (*p*_interaction_ 0.057), MACE(*p*_interaction_ 0.078), and major bleeding patients (*p*_interaction_ 0.078).

## 4 DISCUSSION

In this report of two multinational regional registries from the Asia‐Pacific and Europe, our principal findings are as follows: (i) scAF was more common among Asians than in Europeans, with comorbidities of DM, hypertension and prior ischemic stroke specifically increasing the likelihood of being scAF in Asians; (ii) Although scAF was not associated with the use of OAC, patients with scAF were more likely to be prescribed VKAs and this tendency was more prominent in Asians; (iii) Patients with scAF were less likely to receive rhythm control treatment, without ethnic differences observed; (iv) The scAF was associated with a reduced risk of adverse outcomes, including all‐cause death and MACE, without evidence of ethnic variation.

The major finding of our current comparison is that the prevalence of scAF was higher in Asians than in Europeans (93.6% vs. 80.7%, *p* < 0.01) and this remained true on logistic regression and adjusting for confounders. This is of clinical significance, as patients with scAF tend to seek medical treatment later or tend to ignore treatment suggested. This is likely due to Asians typically not seeking medical attention only when symptoms significantly impact daily activities. This may be due to socio‐economic reasons, especially in countries that do not provide free medical cover or insurance for the whole population. There is also a preference to avoid seeing doctors unless absolutely necessary in Asia. Besides, inherent anthropometric differences may be a significant factor. Recent evidence suggests that chest dimensions influence AF perception, with patients having a narrow antero‐posterior thoracic diameter more likely to be symptomatic.^16^ Caucasians are reported to have wider chests than Asians, which may help explain why Asians are less likely to present with symptoms.^17^

Consistent with previous studies focused only on asymptomatic AF,^10^, ^11^, ^18^ we found that advanced age was associated with a higher likelihood of scAF, while female sex and paroxysmal AF were linked to a lower likelihood of being scAF across the entire population. Racial differences in comorbidities related to scAF were also observed. Previous studies have found that DM, hypertension, and ischemic stroke were more prevalent among asymptomatic patients.^10^, ^11^, ^19^, ^20^ In our analysis, these factors were significantly associated with scAF only in the Asian population, suggesting that Asians with comorbid DM, hypertension, or prior ischemic stroke are likely to experience less pronounced symptoms of AF. Given the high prevalence of these chronic risk factors and the scAF among Asian,^21^, ^22^ it is crucial to emphasise the importance of AF screening for high‐risk populations, especially in Asia.^23^, ^24^

Ethnic differences in clinical characteristics may reflect the complex interplay between prevalent DM, hypertension and ischemic stroke, as well as the impact on associated AF‐related complications (such as stroke and bleeding).^25^, ^26^ DM increases the likelihood of scAF, potentially due to diabetic neuropathy,^27^ which might mask the symptoms. In our study, a higher proportion of Asians with DM were observed compared to Europeans, possibly magnifying the impact of DM on scAF. Hypertension has been shown to further contribute to the development of AF in diabetic patients.^28^ Since both DM and hypertension are risk factors for ischemic stroke,^29^ the relationship between ischemic stroke and scAF may also be influenced by their presence. Moreover, inadequate management of these chronic conditions in Asians may exacerbate the ethnic differences in clinical characteristics.^21^, ^30^ Therefore, these chronic conditions should be viewed as part of a complex syndrome rather than isolated risk factors, highlighting the need for a more comprehensive holistic or integrated care approach to AF management, which is advocated in guidelines^31^, ^32^, ^33^ given the evidence‐based impact on clinical outcomes.^34^, ^35^

In our study, there was no significant link between OAC use and scAF in both Asians and Europeans. This could be attributed to the common practice of determining anticoagulation therapy based on thromboembolism risk, typically assessed using the CHA_2_DS_2_‐VASc score, which is routinely applied in both European and Asian clinical settings. However, we observed a prescription preference for VKA associated with scAF, possibly because the patients maintained good quality of life on VKAs. Notably, the association was more pronounced in Asians, and the choice of NOAC type also varied between Europeans and Asians, potentially reflecting regional disparities in health policies and accessibility of healthcare services.

The lower use of rhythm control treatment associated with scAF is perhaps not surprising as such patients are less inclined to continue antiarrhythmic therapy and may be reluctant to undergo cardioversion or ablation. Earlier studies suggested limited benefits from rhythm control,^36^, ^37^ which may have influenced the doctor's decision for rhythm control. However, more recent research has shown that early detection of AF together with early treatment, regardless of severity of symptoms, could prevent its progression to permanent AF and reduce the risk of cardiovascular death and stroke.^6^ Since the benefits of early rhythm control treatment extend beyond symptom relief, it is essential to reassess its role in scAF patients, considering its potential to improve long‐term outcomes.^6^

While the clinical prognosis of asymptomatic AF has been extensively explored, the prognosis of scAF remains largely unknown. Progression to permanent AF and ischemic stroke was reported to be significantly higher in asymptomatic AF patients compared to those with sAF in the Belgrade AF study.^19^ Mortality at 1 year was more than twice as high in the asymptomatic AF patients in the EORP‐AF pilot registry.^18^ All‐cause mortality was also higher in asymptomatic than symptomatic patients but only in the paroxysmal AF group.^10^ It has been suggested that asymptomatic AF could have a less favourable prognosis than sAF, owing to later detection, lower likelihood for treatment and increased rates of progression to more advanced forms of AF. Other evidence from several meta‐analyses has suggested that the risk of major clinical outcomes, including all‐cause mortality, thromboembolism, stroke and myocardial infarction, did not significantly differ between individuals with and without AF‐related symptoms.^9^, ^38^ Our investigation found that scAF is associated with better clinical outcomes, which may be attributed to differences in study population. Specifically, scAF patients in our analysis were younger and had a lower cardiovascular burden at baseline compared to those with sAF. Additionally, the progression in detection and treatment of AF may have contributed to the improved outcomes observed.

Ethnic differences were not observed with regard to the prognosis related to scAF between Asians and Europeans; however, these findings should be interpreted with caution as given the disparity in sample size between the two cohorts, the results might be largely driven by the larger European cohort. The associations between scAF and adverse clinical outcomes might be underestimated due to limited statistical power. Furthermore, the associations between scAF and adverse clinical outcomes may be confounded by coexisting health conditions. In the EORP cohort, the burden of concurrent diseases might be heavier for patients with sAF than those with scAF, as the probability of scAF decreased with increasing CHA_2_DS_2_‐VASc score. Thus, the worse prognosis associated with sAF in the EORP cohort may be partly attributable to their elevated cardiovascular risk profile. In the APHRS cohort, the prevalences of HF and paroxysmal AF were higher among patients with sAF than those with scAF, potentially conferring additional cardiovascular risk. Meanwhile, Asians with scAF showed higher rates of diabetes, hypertension, and stroke, which may diminish the prognostic differences between scAF and sAF.

When comparing patients with EHRA I to others, asymptomatic AF patients in Asia exhibited significantly higher thromboembolic risks than those with sAF, while this association was not observed among Europeans. Given this notable ethnic disparity, the current symptom‐based clinical pathway may require region‐specific interpretation and adaptation.

The widespread adoption of wearable electrocardiogram monitoring has enabled more frequent early detection of AF patients whose symptoms are not severe enough to impact quality of life.^1^ The guideline‐recommended ABC‐pathway provides limited guidance on clinical decision‐making when symptoms are controlled due to limited clinical evidence. Our findings described the risk profiles and treatment preferences for patients with scAF in Asians and Europeans, indicating that the management of AF should not only depend on the effects of symptoms on their quality of life and prognosis, particularly in the Asian population. As scAF patients in Asia tended to be at high risk, characterised by multiple comorbidities and increased CHA_2_DS_2_‐VASc scores, it highlights the need for holistic or integrated care management including greater innovative efforts for diagnosis and proactive personalised care, given their elderly frail nature with high multimorbidity and polypharmacy leading to clinical complexity.^39^, ^40^, ^41^ Moreover, rhythm control treatment is frequently overlooked among scAF patients, as they are typically perceived as symptom‐controlled and do not require interventions. However, studies have shown that even asymptomatic patients could benefit from rhythm control strategies not only in terms of life quality,^42^ but also clinical prognosis.^43^ Besides, poorly managed uncontrolled AF could lead to morphologic and electrophysiologic remodelling.^44^, ^45^ Therefore, the timely adoption of rhythm control strategy in selected high‐risk scAF patients may prevent AF progression, improve clinical outcomes, and further reduce the economic burden on public health.

### 4.1 Limitations

This is a registry of patients from Asia and Europe and, like all registries, there may be bias in the selection of the patients enrolled in the registry. Second, the social, cultural, and environmental factors could not be fully addressed behind the ethnic differences in our study. Third, while our findings provided valuable insights at the regional level, it was not a direct comparison between ethnic groups. A negligible percentage of patients in the APHRS cohort were from non‐Asian ethnicities, and the EORP cohort also included a minority from other ethnic backgrounds. Furthermore, the APHRS cohort is mainly derived from East Asian countries, with limited representation from Pacific regions. Since we are unable to identify the patients' nationalities, the diversity among Asian countries could not be accounted for. Fourth, the heterogeneity of enrolling sites could not be addressed due to limited site‐specific information. In both cohorts, the treatment decisions were based on the clinical practices of each healthcare institution, which may introduce variability in the implementation of guideline‐based pathways. Fifth, as there is a lack of specificity of AF symptoms, we are unable to distinguish the psychological and other cardiovascular conditions that may be similar to AF symptoms, which might confuse the effects. Finally, the dynamic changes in symptoms over time were not captured in either cohort. Future studies focusing on the effect related to dynamic changes in AF symptoms are required.

## 5 CONCLUSION

Asians and Europeans with scAF demonstrate notable differences in terms of overall prevalence, related risk factors, and clinical management. Asians appear to be more likely to experience scAF, particularly those comorbid with DM, hypertension, and prior ischemic stroke. Despite improved clinical outcomes for patients with scAF, early recognition and a comprehensive approach to managing risk factors could be of essential importance for further prevention of AF progression.

## AUTHOR CONTRIBUTIONS

Wee Siong Teo and Tommaso Bucci were responsible for the conception and design of the study. Manlin Zhao conducted the analysis with the advice from Wee Siong Teo, Tommaso Bucci, and Steven Ho Man Lam. Wee Siong Teo, Manlin Zhao, and Tommaso Bucci were responsible for the interpretation of the data. Wee Siong Teo, Hung‐Fat Tse, Giuseppe Boriani, Tze‐Fan Chao, and Gregory Y. H. Lip were responsible for the acquisition of data and supervised the management of data. Manlin Zhao depicted tables and figures. Wee Siong Teo, Manlin Zhao, and Tommaso Bucci drafted the manuscript. Tommaso Bucci, Hongyu Liu, Yangchen, and Gregory Y. H. Lip reviewed and edited the manuscript. All authors critically appraised the manuscript and approved the final version.

## CONFLICT OF INTEREST STATEMENT

Dr. Lip is a consultant and speaker for Bristol Myers Squibb/Pfizer, Boehringer Ingelheim, Anthos, and Daiichi Sankyo; no fees personally. Dr. Lip is co‐principal investigator of the AFFIRMO project on multimorbidity in AF, which has received funding from the European Union's Horizon 2020 research and innovation program under grant agreement No. 899871. Prof. Boriani reported small speaker fees from Bayer, Boehringer Ingelheim, Boston, Daiichi Sankyo, Janssen, and Sanofi outside of the submitted work. GB is the Principal Investigator of the ARISTOTELES project (Applying ARtificial Intelligence to define clinical trajectorieS for personalised predicTiOn and early deTEction of comorbidity and muLtimorbidity pattErnS) that received funding from the European Union within the Horizon 2020 research and innovation program (Grant N. 101080189). All other authors have reported that they have no relationships relevant to the contents of this paper to disclose.

## Supporting information


Data S1.

## Data Availability

The data underlying this study will be made available upon reasonable request to the corresponding authors.

## References

[1] Linz D , Gawalko M , Betz K , et al. Atrial fibrillation: epidemiology, screening and digital health. Lancet reg Health Eur. 2024;37:100786. 38362546 10.1016/j.lanepe.2023.100786 PMC10866942

[2] Wong CX , Tse HF , Choi EK , et al. The burden of atrial fibrillation in the Asia‐Pacific region. Nat Rev Cardiol. 2024;21:841‐843. 39322762 10.1038/s41569-024-01091-1

[3] Jones J , Stanbury M , Haynes S , et al. Importance and assessment of quality of life in symptomatic permanent atrial fibrillation: patient focus groups from the RATE‐AF trial. Cardiology. 2020;145:666‐675. 32862174 10.1159/000511048

[4] Schnabel RB , Pecen L , Rzayeva N , et al. Symptom burden of atrial fibrillation and its relation to interventions and outcome in Europe. J Am Heart Assoc. 2018;7:e007559. 29776959 10.1161/JAHA.117.007559 PMC6015366

[5] Pastori D , Pignatelli P , Menichelli D , Violi F , Lip GYH . Integrated Care Management of Patients with Atrial Fibrillation and Risk of cardiovascular events: the ABC (atrial fibrillation better care) pathway in the ATHERO‐AF study cohort. Mayo Clin Proc. 2019;94:1261‐1267. 30551910 10.1016/j.mayocp.2018.10.022

[6] Kirchhof P , Camm AJ , Goette A , et al. Early rhythm‐control therapy in patients with atrial fibrillation. N Engl J Med. 2020;383:1305‐1316. 32865375 10.1056/NEJMoa2019422

[7] Vitolo M , Proietti M , Malavasi VL , et al. Adherence to the “atrial fibrillation better care” (ABC) pathway in patients with atrial fibrillation and cancer: A report from the ESC‐EHRA EURObservational research Programme in atrial fibrillation (EORP‐AF) general Long‐term registry. Eur J Intern Med. 2022;105:54‐62. 36028394 10.1016/j.ejim.2022.08.004

[8] Page RL , Wilkinson WE , Clair WK , McCarthy EA , Pritchett EL . Asymptomatic arrhythmias in patients with symptomatic paroxysmal atrial fibrillation and paroxysmal supraventricular tachycardia. Circulation. 1994;89:224‐227. 8281651 10.1161/01.cir.89.1.224

[9] Xiong Q , Proietti M , Senoo K , Lip GY . Asymptomatic versus symptomatic atrial fibrillation: A systematic review of age/gender differences and cardiovascular outcomes. Int J Cardiol. 2015;191:172‐177. 25974193 10.1016/j.ijcard.2015.05.011

[10] Esato M , Chun YH , An Y , et al. Clinical impact of asymptomatic presentation status in patients with paroxysmal and sustained atrial fibrillation: the Fushimi AF registry. Chest. 2017;152:1266‐1275. 28823813 10.1016/j.chest.2017.08.004

[11] Lin J , Wu X‐Y , Long D‐Y , et al. Asymptomatic atrial fibrillation among hospitalized patients: clinical correlates and in‐hospital outcomes in improving care for cardiovascular disease in China‐atrial fibrillation. Europace. 2023;25:euad272. 37712716 10.1093/europace/euad272 PMC10551228

[12] Friberg L , Rosenqvist M , Lindgren A , et al. High prevalence of atrial fibrillation among patients with ischemic stroke. Stroke. 2014;45:2599‐2605. 25034713 10.1161/STROKEAHA.114.006070

[13] Grogan M , Smith HC , Gersh BJ , Wood DL . Left ventricular dysfunction due to atrial fibrillation in patients initially believed to have idiopathic dilated cardiomyopathy. Am J Cardiol. 1992;69:1570‐1573. 1598871 10.1016/0002-9149(92)90705-4

[14] Cox DR . Regression models and life‐tables. J R Stat Soc B Methodol. 2018;34:187‐202.

[15] Stekhoven DJ , Buhlmann P . MissForest–non‐parametric missing value imputation for mixed‐type data. Bioinformatics. 2012;28:112‐118. 22039212 10.1093/bioinformatics/btr597

[16] Sonaglioni A , Grasso E , Nicolosi GL , Lombardo M . Modified Haller index is inversely associated with asymptomatic status in atrial fibrillation patients undergoing electrical cardioversion: a preliminary observation. Minerva Cardiol Angiol. 2024;72:190‐203. 38127440 10.23736/S2724-5683.23.06446-3

[17] Donnelly PM , Yang TS , Peat JK , Woolcock AJ . What factors explain racial differences in lung volumes? Eur Respir J. 1991;4:829‐838. 1955006

[18] Boriani G , Laroche C , Diemberger I , et al. Asymptomatic atrial fibrillation: clinical correlates, management, and outcomes in the EORP‐AF pilot general registry. Am J Med. 2015;128(509):e502. 10.1016/j.amjmed.2014.11.026 25534423

[19] Potpara TS , Polovina MM , Marinkovic JM , Lip GY . A comparison of clinical characteristics and long‐term prognosis in asymptomatic and symptomatic patients with first‐diagnosed atrial fibrillation: the Belgrade atrial fibrillation study. Int J Cardiol. 2013;168:4744‐4749. 23958417 10.1016/j.ijcard.2013.07.234

[20] Bucci T , Nabrdalik K , Shantsila A , et al. Adverse events and clinical correlates in Asian patients with atrial fibrillation and diabetes mellitus: A Report from Asia Pacific Heart Rhythm Society atrial fibrillation registry. J Clin Med. 2024;13:1274. 38592107 10.3390/jcm13051274 PMC10932296

[21] Chia YC , Kario K , Turana Y , et al. Target blood pressure and control status in Asia. J Clin Hypertens (Greenwich). 2020;22:344‐350. 31742891 10.1111/jch.13714 PMC8029870

[22] Sinclair MR , Ardehali M , Diamantidis CJ , Corsino L . The diabetes cardiovascular outcomes trials and racial and ethnic minority enrollment: impact, barriers, and potential solutions. Front Public Health. 2024;12:1412874. 39525461 10.3389/fpubh.2024.1412874 PMC11545964

[23] Chan NY , Orchard J , Agbayani MJ , et al. 2021 Asia Pacific Heart Rhythm Society (APHRS) practice guidance on atrial fibrillation screening. J Arrhythm. 2022;38:31‐49. 35222749 10.1002/joa3.12669 PMC8851593

[24] Chao TF , Yeh YH , Chan YH , et al. The report of community‐based and government‐endorsed screening program of atrial fibrillation in Taiwan. Thromb Haemost. 2024;124:61‐68. 37434320 10.1055/a-2127-0690

[25] Kang DS , Yang PS , Kim D , et al. Racial differences in ischemic and hemorrhagic stroke: An ecological epidemiological study. Thromb Haemost. 2024;124:883‐892. 38423097 10.1055/a-2278-8769

[26] Kang DS , Yang PS , Kim D , et al. Racial differences in bleeding risk: An ecological epidemiological study comparing Korea and United Kingdom subjects. Thromb Haemost. 2024;124:842‐851. 38359877 10.1055/a-2269-1123 PMC11349425

[27] Sugishita K , Shiono E , Sugiyama T , Ashida T . Diabetes influences the cardiac symptoms related to atrial fibrillation. Circ J. 2003;67:835‐838. 14578615 10.1253/circj.67.835

[28] Gumprecht J , Lip GYH , Sokal A , et al. Relationship between diabetes mellitus and atrial fibrillation prevalence in the polish population: a report from the non‐invasive monitoring for early detection of atrial fibrillation (NOMED‐AF) prospective cross‐sectional observational study. Cardiovasc Diabetol. 2021;20:128. 34167520 10.1186/s12933-021-01318-2 PMC8228888

[29] Chen RL , Balami JS , Esiri MM , Chen LK , Buchan AM . Ischemic stroke in the elderly: an overview of evidence. Nat Rev Neurol. 2010;6:256‐265. 20368741 10.1038/nrneurol.2010.36

[30] Islam NS , Kwon SC , Wyatt LC , et al. Disparities in diabetes management in Asian Americans in New York City compared with other racial/ethnic minority groups. Am J Public Health. 2015;105(Suppl 3):S443‐S446. 25905853 10.2105/AJPH.2014.302523 PMC4455523

[31] Chao TF , Joung B , Takahashi Y , et al. 2021 focused update of the 2017 consensus guidelines of the Asia Pacific Heart Rhythm Society (APHRS) on stroke prevention in atrial fibrillation. J Arrhythm. 2021;37:1389‐1426. 34887945 10.1002/joa3.12652 PMC8637102

[32] Wang Y , Guo Y , Qin M , et al. Chinese expert consensus guidelines on the diagnosis and treatment of atrial fibrillation in the elderly, endorsed by geriatric Society of Chinese Medical Association (cardiovascular group) and Chinese Society of Geriatric Health Medicine (cardiovascular branch): executive summary. Thromb Haemost. 2024;124:897‐911. 38744425 10.1055/a-2325-5923 PMC11436293

[33] Potpara T , Romiti GF , Sohns C . The 2024 European Society of Cardiology Guidelines for diagnosis and Management of Atrial Fibrillation: A viewpoint from a practicing Clinician's perspective. Thromb Haemost. 2024;124:1087‐1094. 39374908 10.1055/a-2434-9244

[34] Romiti GF , Guo Y , Corica B , et al. Mobile health‐technology‐integrated Care for Atrial Fibrillation: A win ratio analysis from the mAFA‐II randomized clinical trial. Thromb Haemost. 2023;123:1042‐1048. 37247623 10.1055/s-0043-1769612

[35] Treewaree S , Lip GYH , Krittayaphong R . Non‐vitamin K antagonist Oral anticoagulant, warfarin, and ABC pathway adherence on hierarchical outcomes: win ratio analysis of the COOL‐AF registry. Thromb Haemost. 2024;124:69‐79. 37625457 10.1055/s-0043-1772773

[36] van Gelder IC , Hagens VE , Bosker HA , et al. A comparison of rate control and rhythm control in patients with recurrent persistent atrial fibrillation. N Engl J Med. 2002;347:1834‐1840. 12466507 10.1056/NEJMoa021375

[37] Wyse DG , Waldo AL , DiMarco JP , et al. A comparison of rate control and rhythm control in patients with atrial fibrillation. N Engl J Med. 2002;347:1825‐1833. 12466506 10.1056/NEJMoa021328

[38] Karakasis P , Pamporis K , Siontis KC , et al. Major clinical outcomes in symptomatic vs. asymptomatic atrial fibrillation: a meta‐analysis. Eur Heart J. 2025;46(13):1189‐1202. doi:10.1093/eurheartj/ehae694 39428997

[39] Boriani G , Mei DA , Lip GYH , Consortium A . A European‐multicenter network for the implementation of Artificial intelligence to manage complexity and comorbidities of atrial fibrillation patients: the ARISTOTELES Consortium. Thromb Haemost. 2025;125:189. 39855632 10.1055/a-2508-5708

[40] Ortega‐Martorell S , Olier I , Ohlsson M , Lip GYH , Consortium T . Target: A major European project aiming to advance the personalised Management of Atrial Fibrillation‐Related Stroke via the development of health virtual twins technology and Artificial intelligence. Thromb Haemost. 2025;125:7‐11. 39401519 10.1055/a-2438-5671

[41] Romiti GF , Proietti M , Bonini N , et al. Clinical complexity domains, anticoagulation, and outcomes in patients with atrial fibrillation: A report from the GLORIA‐AF registry phase II and III. Thromb Haemost. 2022;122:2030‐2041. 36037828 10.1055/s-0042-1756355

[42] Mohanty S , Santangeli P , Mohanty P , et al. Catheter ablation of asymptomatic longstanding persistent atrial fibrillation: impact on quality of life, exercise performance, arrhythmia perception, and arrhythmia‐free survival. J Cardiovasc Electrophysiol. 2014;25:1057‐1064. 24903064 10.1111/jce.12467

[43] Kim JY , Park HS , Park HW , et al. Clinical outcomes of rhythm control strategies for asymptomatic atrial fibrillation according to the quality‐of‐life score: the CODE‐AF (comparison study of drugs for symptom control and complication prevention of atrial fibrillation) registry. J Am Heart Assoc. 2022;11:e025956. 36073646 10.1161/JAHA.122.025956 PMC9683675

[44] Wijffels MC , Kirchhof CJ , Dorland R , Allessie MA . Atrial fibrillation begets atrial fibrillation. A study in awake chronically instrumented goats. Circulation. 1995;92:1954‐1968. 7671380 10.1161/01.cir.92.7.1954

[45] Eckstein J , Verheule S , de Groot NM , Allessie M , Schotten U . Mechanisms of perpetuation of atrial fibrillation in chronically dilated atria. Prog Biophys Mol Biol. 2008;97:435‐451. 18378284 10.1016/j.pbiomolbio.2008.02.019

