# Supplementary material for: Clinical associations and prognosis in Asian and European patients with symptom‐controlled atrial fibrillation: Insights from two prospective registries in Europe and Asia
Source: Eur J Clin Invest. 2025 Jun 9;55(11):e70086. doi: 10.1111/eci.70086 (PMC12517246; doi:10.1111/eci.70086)
Supplement: Supplementary file 1 — Data S1. [file ECI-55-e70086-s001.docx]

**Clinical associations and prognosis in Asian and European patients with symptom-controlled atrial fibrillation:** **Insights from two prospective registries in Europe and Asia**

Wee Siong Teo^1^†, Manlin Zhao^2,3^ †, Tommaso Bucci^2,4^†, Steven Ho Man Lam^2,5^, Hongyu Liu^2,6^, Yang Chen^2,7^, Giuseppe Boriani^8^, Tze-Fan Chao^9,10^,

Gregory Y.H. Lip^2,11,12^ *

Supplementary material

**Table S1. Baseline characteristics and differences between Europeans and Asians.**

|  | EORP (N=9620) | APHRS (N=3957) | P value |
| --- | --- | --- | --- |
| Prevalence of scAF, n (%) | 7768 (80.7) | 3702 (93.6) | <0.001 |
| Age (years) [IQR] | 71.0 [63.0, 77.0] | 69.0 [61.0, 77.0] | <0.001 |
| Age≥65 years, n (%) | 6693 (69.6) | 2595 (65.6) | <0.001 |
| Female, n (%) | 3870 (40.2) | 1382 (34.9) | <0.001 |
| BMI (kg/m^2^) [IQR] | 27.6 [24.8, 31.2] | 24.7 [22.3, 27.3] | <0.001 |
| HF, n (%) | 3694 (38.7) | 845 (21.6) | <0.001 |
| CAD, n (%) | 2492 (27.4) | 758 (19.5) | <0.001 |
| Hypertension, n (%) | 5861 (61.5) | 2414 (61.4) | 0.951 |
| Diabetes, n (%) | 2180 (22.8) | 951 (24.4) | 0.048 |
| Lipid disorder, n (%) | 3786 (41.1) | 1503 (38.6) | 0.008 |
| Thromboembolism events, n (%) | 1116 (11.7) | 439 (11.2) | 0.423 |
| CKD, n (%) | 1167 (12.2) | 300 (7.6) | <0.001 |
| Liver disease, n (%) | 266 (2.8) | 178 (4.5) | <0.001 |
| COPD, n (%) | 852 (8.9) | 110 (2.8) | <0.001 |
| Dementia, n (%) | 130 (1.4) | 73 (1.9) | 0.039 |
| Cancer, n (%) | 192 (2.0) | 87 (2.2) | 0.510 |
| Paroxysmal AF, n (%) | 2422 (25.6) | 1670 (42.3) | <0.001 |
| Persistent AF, n (%) | 2342 (24.8) | 1292 (32.8) | <0.001 |
| CHA_2_DS_2_-VaSc score [IQR] | 3.0 [2.0, 4.0] | 3.0 [1.0, 4.0] | <0.001 |
| CHA_2_DS_2_-VaSc≥2, n (%) | 7718 (80.3) | 2899 (73.3) | <0.001 |
| HAS-BLED score [IQR] | 1.0 [1.0, 2.0] | 1.0 [1.0, 2.0] | <0.001 |
| HAS-BLED ≥3, n (%) | 1656 (17.2) | 536 (13.5) | <0.001 |
| OACs, n (%) | 8312 (86.4) | 3271 (82.7) | <0.001 |
| VKAs, n (%) | 4778 (49.7) | 806 (20.4) | <0.001 |
| NOACs, n (%) | 3541 (36.8) | 2465 (62.3) | <0.001 |
| EHRA score, n (%) |  |  | <0.001 |
| EHRA I | 4349 (45.2) | 2531 (64.0) |  |
| EHRA II | 3419 (35.5) | 1171 (29.6) |  |
| EHRA III | 1635 (17.0) | 226 (5.7) |  |
| EHRA IV | 217 (2.3) | 29 (0.7) |  |

AF=atrial fibrillation, scAF= symptom-controlled atrial fibrillation, BMI=body mass index, HF= heart failure, CAD= coronary artery disease, CKD= chronic kidney disease, COPD= chronic obstructive pulmonary disease, OAC= oral anticoagulants, VKA= vitamin K antagonists, NOAC= novel oral anticoagulants.

**Table S2. Baseline characteristics and differences between scAF and sAF.**

|  | **sAF (N=2107)** | **scAF (N=11470)** | **P value** |
| --- | --- | --- | --- |
| Asians, n (%) | 255 (12.1) | 3702 (32.3) | <0.001 |
| Age (years) [IQR] | 69.0 [61.0, 77.0] | 70.0 [62.0, 77.0] | 0.001 |
| Age≥65 years, n (%) | 1406 (66.8) | 7882 (68.7) | 0.076 |
| Female, n (%) | 1014 (48.1) | 4238 (36.9) | <0.001 |
| BMI (kg/m^2^) [IQR] | 27.2 [24.2, 31.2] | 26.6 [24.0, 30.1] | <0.001 |
| HF, n (%) | 966 (46.1) | 3573 (31.5) | <0.001 |
| CAD, n (%) | 538 (27.4) | 2712 (24.6) | 0.009 |
| Hypertension, n (%) | 1282 (61.5) | 6993 (61.4) | 0.987 |
| Diabetes, n (%) | 483 (23.1) | 2648 (23.3) | 0.833 |
| Lipid disorder, n (%) | 834 (41.5) | 4455 (40.2) | 0.296 |
| Thromboembolism events, n (%) | 241 (11.6) | 1314 (11.6) | 0.958 |
| CKD, n (%) | 298 (14.2) | 1169 (10.3) | <0.001 |
| Liver disease, n (%) | 89 (4.3) | 355 (3.1) | 0.008 |
| COPD, n (%) | 201 (9.6) | 761 (6.7) | <0.001 |
| Dementia, n (%) | 47 (2.2) | 156 (1.4) | 0.003 |
| Cancer, n (%) | 43 (2.1) | 236 (2.1) | 1.000 |
| Paroxysmal AF, n (%) | 630 (30.2) | 3462 (30.6) | 0.737 |
| Persistent AF, n (%) | 608 (29.2) | 3026 (26.8) | 0.025 |
| CHA_2_DS_2_-VaSc score [IQR] | 3.0 [2.0, 4.0] | 3.0 [2.0, 4.0] | <0.001 |
| CHA_2_DS_2_-VaSc≥2, n (%) | 1688 (80.1) | 8929 (77.9) | 0.03 |
| HAS-BLED score [IQR] | 1.0 [1.0, 2.0] | 1.0 [1.0, 2.0] | 0.206 |
| HAS-BLED ≥3, n (%) | 378 (17.9) | 1814 (15.8) | 0.02 |
| OACs, n (%) | 1802 (85.5) | 9781 (85.3) | 0.79 |
| VKAs, n (%) | 932 (44.2) | 4652 (40.6) | 0.002 |
| NOACs, n (%) | 872 (41.4) | 5134 (44.8) | 0.004 |
| Rhythm control strategy at discharge, n (%) | 1004(47.7) | 3926(34.2) | <0.001 |
| Catheter ablation, n (%) | 256(22.0) | 820(13.3) | <0.001 |
| Electrical cardioversion, n (%) | 441(37.9) | 1301(21.1) | <0.001 |
| AAD prescription at discharge, n (%) | 739(35.1) | 2773(24.2) | <0.001 |

AF=atrial fibrillation, scAF= symptom-controlled atrial fibrillation, sAF= symptom atrial fibrillation, BMI=body mass index, HF= heart failure, CAD= coronary artery disease, CKD= chronic kidney disease, COPD= chronic obstructive pulmonary disease, OAC= oral anticoagulants, VKA= vitamin K antagonists, NOAC= novel oral anticoagulants.

**Table S3. Ethnic differences (Asians vs. Europeans) in terms of prevalence of symptom-controlled AF.**

adjusted for: age, sex, paroxysmal AF, heart failure, diabetes, hypertension, vascular diseases, chronic kidney disease, ischemic stroke, cancer.

|  | **OR** | **95%CI** | **p value** |
| --- | --- | --- | --- |
| **Symptom-controlled AF** | 3.19 | 2.78-3.68 | <0.001 |

AF= atrial fibrillation, OR= Odd ratio, CI= Confidence interval.

**Table S4. Adjusted logistic regression analysis investigating the association between symptom-controlled AF and different types of NOAC.**

Logistic models among patients taking NOAC were adjusted for age, sex, paroxysmal AF, HF, DM, hypertension, vascular diseases, chronic kidney disease, ischemic stroke, cancer, ethnicity. Logistic models performed in EORP and APHRS cohort separately were adjusted for age, sex, paroxysmal AF, heart failure, DM, hypertension, vascular diseases, chronic kidney disease, ischemic stroke, cancer.

| **NOAC type** | **OR (95%CI)** | **P-inter** |
| --- | --- | --- |
| Dabigatran |  | 0.096 |
| APHRS | 1.11(0.75-1.70) |  |
| EORP | 0.81(0.67-1.00) |  |
| Overall | 0.89(0.74-1.06) |  |
| Rivaroxaban |  | 0.389 |
| APHRS | 0.99(0.72-1.37) |  |
| EORP | 1.17(0.99-1.39) |  |
| Overall | 1.12(0.97-1.31) |  |
| Apixaban |  | 0.060 |
| APHRS | 1.33(0.94-1.92) |  |
| EORP | 0.87(0.73-1.06) |  |
| Overall | 0.97(0.82-1.14) |  |
| Edoxaban |  | <0.001 |
| APHRS | 0.65(0.45-0.95) |  |
| EORP | 5.09(2.10-16.74) |  |
| Overall | 1.04(0.76-1.46) |  |

**Table S5. Results of univariable cox regression analysis for the relationship between symptom-controlled AF and outcomes of the study.**

|  | HR | 95%CI | P Value | P-inter | |
| --- | --- | --- | --- | --- | --- |
| Composite outcomes | | | | |  |
| APHRS | 0.84 | 0.47-1.52 | 0.567 | 0.769 | |
| EORP | 0.77 | 0.68-0.88 | <0.001 |  |  |
| Overall | 0.72 | 0.63-0.81 | <0.001 |  |  |
| All-cause death | | | | |  |
| APHRS | 0.80 | 0.41-1.58 | 0.522 | 0.886 | |
| EORP | 0.76 | 0.65-0.88 | <0.001 |  |  |
| Overall | 0.71 | 0.61-0.82 | <0.001 |  |  |
| MACE | | | | |  |
| APHRS | 0.96 | 0.39-2.38 | 0.931 | 0.564 | |
| EORP | 0.73 | 0.62-0.87 | <0.001 |  |  |
| Overall | 0.68 | 0.57-0.80 | <0.001 |  |  |
| ACS | | | | |  |
| APHRS | 0.50 | 0.15-1.67 | 0.259 | 0.403 | |
| EORP | 0.86 | 0.63-1.16 | 0.315 |  |  |
| Overall | 0.76 | 0.57-1.02 | 0.071 |  |  |
| Cardiovascular death | | | | |  |
| APHRS | 1.68 | 0.23-12.38 | 0.613 | 0.361 | |
| EORP | 0.65 | 0.51- 0.82 | <0.001 |  |  |
| Overall | 0.59 | 0.46-0.75 | <0.001 |  |  |
| Any TE | | | | |  |
| APHRS | 1.85 | 0.25-13.64 | 0.545 | 0.348 | |
| EORP | 0.71 | 0.52-0.96 | 0.029 |  |  |
| Overall | 0.69 | 0.51-0.93 | 0.016 |  |  |
| Major bleeding | | | | |  |
| APHRS | 0.58 | 0.23-1.46 | 0.245 | 0.257 | |
| EORP | 1.02 | 0.71-1.46 | 0.912 |  |  |
| Overall | 0.94 | 0.67-1.30 | 0.693 |  |  |

**Table S6. Results of multivariable cox regression analysis for the relationship between asymptomatic AF and outcomes of the study.**

|  | HR | 95%CI | P Value | P- inter | |
| --- | --- | --- | --- | --- | --- |
| **Composite outcomes** | | | | |  |
| APHRS | 1.33 | 0.92-1.92 | 0.131 | 0.057 | |
| EORP | 0.99 | 0.89-1.11 | 0.886 |  |  |
| Overall | 0.97 | 0.88-1.08 | 0.600 |  |  |
| **All-cause death** | | | | |  |
| APHRS | 1.34 | 0.86-2.09 | 0.203 | 0.183 | |
| EORP | 1.08 | 0.95-1.23 | 0.265 |  |  |
| Overall | 1.05 | 0.93-1.19 | 0.450 |  |  |
| **MACE** | | | | |  |
| APHRS | 1.58 | 0.92-2.73 | 0.098 | 0.078 | |
| EORP | 0.97 | 0.83-1.12 | 0.653 |  |  |
| Overall | 0.95 | 0.83-1.09 | 0.478 |  |  |
| **ACS** | | | | |  |
| APHRS | 0.84 | 0.37-1.92 | 0.681 | 0.885 | |
| EORP | 0.90 | 0.70-1.17 | 0.434 |  |  |
| Overall | 0.85 | 0.67-1.09 | 0.203 |  |  |
| **Cardiovascular death** | | | | |  |
| APHRS | 2.38 | 0.80-7.11 | 0.120 | 0.186 | |
| EORP | 1.11 | 0.89-1.37 | 0.358 |  |  |
| Overall | 1.06 | 0.86-1.30 | 0.589 |  |  |
| **Any TE** | | | | |  |
| APHRS | 3.03 | 1.03-8.90 | 0.044 | 0.024 | |
| EORP | 0.90 | 0.68-1.18 | 0.435 |  |  |
| Overall | 0.95 | 0.74-1.23 | 0.700 |  |  |
| **Major bleeding** | | | | |  |
| APHRS | 1.59 | 0.79-3.19 | 0.191 | 0.078 | |
| EORP | 0.88 | 0.66-1.17 | 0.382 |  |  |
| Overall | 0.96 | 0.75-1.24 | 0.769 |  |  |
